# Entropy in Brain Networks

**DOI:** 10.3390/e23091157

**Published:** 2021-09-02

**Authors:** Jesús Poza, María García, Javier Gomez-Pilar

**Affiliations:** 1 Biomedical Engineering Group, University of Valladolid, 47011 Valladolid, Spain; maria.garcia@tel.uva.es (M.G.); javier.gomez@gib.tel.uva.es (J.G.-P.); 2 Centro de Investigación Biomédica en Red en Bioingeniería, Biomateriales y Nanomedicina (CIBER-BBN), 47011 Valladolid, Spain; 3 Instituto de Investigación en Matemáticas—IMUVA, University of Valladolid, 47001 Valladolid, Spain

A thorough and comprehensive understanding of the human brain ultimately depends on knowledge of large-scale brain organization. Although it has long been assumed that specific functions are attributable to local and isolated brain areas, a number of advances in neuroimaging have shown accumulating evidence supporting that brain behavior is highly dependent on the dynamic interactions of distributed brain areas, operating as large-scale networks [1,2,3]. These networks, both structural and functional, have been identified through a series of imaging modalities with a common characteristic: a complex, hierarchical, and dynamic tangle of neural connections governed by intricate nonlinear interactions at different levels. The unraveling of the complex structure and the functional organization of the brain is not obvious, being necessary to consider the nonlinear nature of such a system. In this context, knowing that tools from information theory are well-suited for this task, this Special Issue was intended to shed some light on the comprehension of brain networks by using entropy-related measures. Hence, the combination of network analysis and information theory provides a theoretical and operational framework for obtaining both quantitative and qualitative descriptions of the intrinsic properties of human brain.

This Special Issue is dedicated to providing solutions and delving into concepts, methods, and algorithms for improving the characterization of brain networks both in healthy subjects and/or in diverse disorders affecting the central nervous system. The nine articles included in this Special Issue propose and discuss new tools derived from the information theory field to investigate brain behavior. They illustrate the potential and pertinence of this tool to gain further knowledge into brain functioning. This is the case of the study by Alù et al. [4], in which they assessed the reproducibility and stability of approximate entropy across time, as well as hemispheric differences, in a longitudinal electroencephalographic (EEG) dataset. From a more theoretical point of view, Maren [5] addressed a closely related concept to entropy: enthalpy, which is used to characterize 2D image topographies using the 2D Cluster Variation Method (CVM). Specifically, activation and interaction enthalpy parameters were associated with characteristic configuration variable values of the resulting pattern from free energy minimization of 2D CVM. The versatility of measures from information theory is also evident in the study carried out by Melin et al. [6], in which entropy-based construct specification equations are used to improve validity of memory tests and to design novel combined tests. This new methodology can indeed contribute to obtain reliable diagnoses of neurological diseases, such as dementia, and to properly characterize cognitive processes generated by cerebral networks by providing accurate scoring.

Entropy-related metrics have also shown their potential to gain knowledge on diverse brain diseases. Due to the complexity of the problem, multidisciplinarity is clear in most of these works. This is the case of the study carried out by Revilla-Vallejo and colleagues [7]. They propose a new methodology based on analyzing the distribution of the weights of the brain network from EEG after source reconstruction to improve understanding of the neural mechanisms associated with the progression of Alzheimer disease (AD). Wang et al. [8] also want to delve into the AD progression from the functional connectivity point of view. Making use of functional resonance imaging (fMRI) and network entropy, microcanonical and canonical ensembles are applied to describe the altered macroscopic properties of the brain network due to AD. A different entropy-based measure of signal irregularity, conditional entropy of ordinal patterns (CEOP), was evaluated as a tool for the diagnosis of epileptic patients by Liu and Fu [9]. They assessed CEOP using both simulated EEG recordings generated by means of the neural mass model and a real EEG database. Their results showed the potential of this entropic measure as a novel recognition strategy of epilepsy based on EEG recordings.

The measures derived from information theory, and in particular those related to entropy, have also exhibited their ability to unravel relevant information from underlying brain networks in different conditions. In this regard, the combination of measures from information theory with both structural and functional connectivity analyses yields a new framework to further understand brain organization. This can be observed in the work of Jao et al. [10], in which irregularity of the network anatomical structure, in particular inter- and intra-lobe brain connectivity, was analyzed using the fractal dimension. Their results suggested that aging induces morphological changes in the structural brain network generated from magnetic resonance imaging (MRI), which could be related to rewiring and reconfiguring modules as compensatory mechanisms for the functional deficit in inter- and intra-lobe brain connectivity. From a different perspective, Zhang et al. [11] focused on analyzing driver alertness while listening to the radio by analyzing functional coupling. To characterize driver fatigue, they explored the relationships between differential entropy and functional connectivity networks generated from EEG recordings. Being aware of the importance of the connectivity metric used in each study, far from being standardized, Fraschini and colleagues [12] compared different functional connectivity measures. They concluded that source-level EEG connectivity results and the associated network measures exhibit a certain variability that depends on the metric used to quantify functional coupling. Further methodological approaches are then required to obtain robust connectivity measures. In this regard, measures from information theory can play a relevant role in reducing the uncertainly and ambiguity in the connectivity patterns and obtaining a proper estimate of functional coupling.

The brain is presumably the most intricate and complex biological system. Its comprehension is an open challenge that requires new computational tools and methodological approaches. Information theory methods, and particularly entropy, provide a versatile and flexible framework with the potential to move neuroscience research forward. In this Special Issue, entropy-related approaches were applied to further understand functional and structural organization of brain networks. They provide relevant pieces of research that addressed timely, current neuroscience topics. It is our hope that the reader will enjoy the articles included in the Special Issue and will find them helpful and worthy contributions to current neuroscience research.
